# Complement in anti-glomerular basement membrane glomerulonephritis

**DOI:** 10.3389/fimmu.2025.1442955

**Published:** 2025-05-21

**Authors:** Pei Zhang, Kai-li Shi, Chun-lin Gao, Feng Xu, Li-li Jia, Ji-chao Sun

**Affiliations:** ^1^ Department of Pediatrics, Jinling Hospital, Affiliated Hospital of Medical School, Nanjing University, Nanjing, China; ^2^ Department of Pediatrics, Jinling Hospital, Nanjing Medical University, Nanjing, China; ^3^ National Clinical Research Center for Kidney Disease, Jinling Hospital, Nanjing Medical University, Nanjing, China; ^4^ Department of Pediatrics, The First Affiliated Hospital of Guangxi University of Chinese Medicine, Nanning, China

**Keywords:** anti-glomerular basement membrane, glomerulonephritis, complement, C3, C1q

## Abstract

Anti-glomerular basement membrane glomerulonephritis (anti-GBM GN) is a rare autoimmune disease that often progresses to end-stage renal disease (ESRD). Complement activation and anti-GBM GN are closely related, as evidenced by the renal pathological characteristics of patients with anti-GBM GN, which include the linear deposition of immunoglobulin G (IgG) and C3 along the GBM. Increasing evidence suggests that all three pathways of complement activation may be involved in the pathogenesis and progression of anti-GBM GN. Anti-GBM GN’s clinical symptoms are linked to complement-related proteins, which are risk factors that impact the disease’s prognosis. This suggests that complement activation and activity may be the primary causes of renal damage in anti-GBM GN. Therefore, biomarkers of complement activation can identify anti-GBM GN cases that may progress to severe renal damage, and complement inhibition may become a new strategy for the clinical treatment of anti-GBM GN.

## Introduction

Anti-glomerular basement membrane (anti-GBM) disease is a rare autoimmune disorder in which the target antigen is located within the non-collagenous domain of the alpha-3 chain of type IV collagen [α3(IV)NC1]. Blood contains anti-GBM antibodies, which can accumulate in the kidneys and/or lungs and cause rapid progressive glomerulonephritis (RPGN) and pulmonary hemorrhage. Because the alveoli and glomeruli share basement membrane antigens, the kidneys and lungs are the primary organs affected. In the revised International Chapel Hill Consensus Conference vasculitis nomenclature, anti-GBM disease is classified as an immune complex small vessel vasculitis (1). Lung involvement presents as diffuse alveolar hemorrhage, and simultaneous lung and kidney damage is known as Goodpasture’s syndrome. Kidney involvement is referred to as anti-GBM glomerulonephritis (GN). The overall incidence of anti-GBM disease is 0.5–1 per million (2–4), with the precise incidence in children still unclear. Anti-GBM GN is a rapidly progressive crescentic GN, with approximately 80% of patients showing crescent formation in over half of the glomeruli (5). In patients undergoing kidney biopsy, anti-GBM disease accounts for 1%–5% of GN cases and 10%–15% of crescentic GN cases (6, 7). There are 40%–60% of patients present with Goodpasture’s syndrome (8). Isolated anti-GBM GN has a poor prognosis, high rates of morbidity and mortality, and no discernible gender or age differences in occurrence. Nearly all individuals develop end-stage renal disease (ESRD) if therapy is delayed.

The renal pathology characteristics of patients with anti-GBM GN include crescent formation, Bowman capsule rupture, segmental necrosis of the glomerular tuft, and linear deposits of immunoglobulin G (IgG) and C3 along the GBM, indicating a close relationship between antigen–antibody reactions, complement activation, and disease pathogenesis. Increasing evidence suggests that all three pathways of complement system activation are involved in the development of anti-GBM GN. In this review, we provide the latest preclinical and clinical evidence on the role of complement activation in anti-GBM GN, aiming to offer new potential therapeutic strategies for the clinical treatment of anti-GBM GN.

## The complement system

The complement system was first described in the 19th century. It is considered an ancient protein-based defense mechanism and is part of the innate immune system. There are three main activation pathways of the complement system: the classical pathway (CP), the alternative pathway (AP), and the lectin pathway (LP), although cathepsin, proteolytic, and intracellular complement activation pathways are receiving more and more attention as new routes to trigger complement activation. For the purposes of this review, we will focus on the more established pathways. Components involved in the CP include C1, C4, and C2; components involved in the LP include MBL and serine proteases; components involved in the AP include factor B, factor D, and properdin, among others. These three pathways converge at C3 and ultimately lead to the formation of the membrane attack complex (MAC) C5b-9. Complement activation mainly produces three types of effector molecules: (1) anaphylatoxins, such as C3a and C5a, which interact with their respective G protein-coupled receptors (C3a receptor and C5a receptor) to attract and stimulate inflammatory cells to release inflammatory substances; (2) opsonins, including C3b, iC3b, and C3d, which primarily facilitate the movement and elimination of target cells and immune complexes through covalent attachment; and (3) C5b-9, which can directly destroy pathogens or damaged cells. The kidney is uniquely susceptible to complement-mediated damage for several reasons (9): (1) The kidney is one of the major organs outside the liver for synthesizing complement proteins. The epithelial cells of the glomerulus, mesangial cells, and epithelial cells of the renal tubules can all synthesize and secrete complement proteins such as C3 and C4. (2) A redundant system of soluble and membrane-bound regulators typically prevents uncontrolled complement activation on host cells. However, there is a lack of membrane-bound regulators in GBM, which is dependent on circulating soluble complement regulators [principally factor H (FH) and C4b binding protein]. Moreover, the FH co-localizes with collagen IV alpha 3 (COLIV alpha 3), indicating the important role of the FH in protecting the GBM. (3) The glomerulus is directly exposed to circulating immune effector molecules, making it susceptible to immune complex deposition, including immunoglobulins and complement components along the GBM.

## Complement pathway in animal models of anti-GBM GN

The renal pathological features of wild-type anti-GBM GN animals include neutrophil infiltration, glomerular capillary thrombosis, proteinuria, and deposition of C3 and C4 in the glomeruli. Compared to C3-deficient and C4-deficient mice, wild-type mice exhibit more pronounced early neutrophil infiltration into the glomeruli. In C3- and C4-deficient mice, the absence of complement expression not only inhibits the progression of proteinuria but also reduces glomerular capillary thrombosis. Moreover, the renal protective effect of C3 deficiency is greater than C4 (10). However, researchers have reported that in the autologous phase of the same model, proteinuria and uremia are more severe in C3- and C1q-deficient mice, possibly due to impaired immune complex clearance mediated by C1q and C3 (11, 12). The same study also found that using cobra venom factor (CVF) to stimulate anti-GBM GN WKY rats resulted in the depletion of plasma complement on the third day. Regarding proteinuria and the proportion of crescents, there were no appreciable variations between the CVF treatment group and the control group. This may be related to the construction of the animal model, the type of experimental animals, and the titer of anti-GBM antibodies (13).

Otten et al. (14). confirmed that the mice deficient in the CP (C1q/mice) or the CP and LP (C4/mice) exhibit almost the same degree of proteinuria, while the degree of proteinuria in C3/mice is significantly reduced. In the kidneys of C1q/and C4/mice, C3 deposition remains detectable, whereas C1q and C4 deposition is diminished or absent. This indicates that in anti-GBM GN, complement activation can shift from the CP to the AP. The standard complement route is first triggered by anti-GBM antibodies, while the AP subsequently increases the synthesis of inflammatory molecules (15, 16). This also explains why C1q/mice and C4/mice maintain high levels of proteinuria.

Properdin is an important positive regulator. It can extend the half-life of C3bBb by 10 times, thereby enhancing the cleavage of C3 (17). Recent studies on anti-GBM GN have demonstrated significant deposition of properdin and other AP components in human glomeruli affected by anti-GBM antibodies. Results of time-course immunohistochemistry show that the deposition of C1q, C3, and properdin in anti-GBM is consistent with serum levels, suggesting complement activation mediated by the CP and AP. Moreover, 48 h after treatment with anti-GBM antibodies, the mice showed enhanced C3 staining, which co-localized with properdin. Deposition of C6 and C9 is only observed significantly 24 h after administration and continues to increase within 48 to 72 h, consistent with the increase in C3 and properdin deposition. This indicates that complement-mediated injury in the anti-GBM GN may weaken within 48 to 72 h (18).

The role of the complement in different stages of anti-GBM disease is contradictory. During the acute phase of anti-GBM GN, injury from inflammation is confirmed to be complement-dependent, triggered by the binding of heterologous antibodies to the GBM. Mice deficient in C3 exhibit milder renal damage. In contrast, during the autologous phase, the immune response is mediated by antibodies targeting antigens fixed within the GBM and is largely complement-independent (12).

## Complement pathway in humans of anti-GBM GN

C3 is the most common complement deposition in anti-GBM GN. C3 deposition can be observed in 41%–69.2% of the GBM of patients with anti-GBM GN (19–21), while the decrease in serum C3 levels is present in only 6%–27.7% of patients (20, 21). C3 activation fragments are the most commonly detected complement proteins deposited in anti-GBM GN. According to the literature, the kidney mesangial (22, 23), glomerular epithelial (22), endothelial (24), and renal tubule cells (25) can produce C3. Locally synthesized C3 may contribute to the pathogenesis of kidney injury, with a function distinct from that of circulating complement (11). Research indicates that serum C3 levels and renal C3 staining intensity are independent predictors of renal prognosis in anti-GBM GN (19, 21). C3 deposition can also promote T-cell expansion (26), and in patients with anti-GBM GN, T-cell infiltration is associated with renal damage (27, 28) and poor renal survival (29).

C1q deposition in anti-GBM GN is not common; it can be observed in 3%–16.7% of the GBM in anti-GBM GN (19, 20). Anti-C1q antibodies (a-C1q Abs) have been confirmed to promote the deposition of C1q on target organs and cell surfaces. The plasma a-C1qAb positivity rate in anti-GBM GN is 45.45%–63.64% (30, 31). Currently, no association has been found between glomerular C1q deposition and the severity of kidney injury or disease prognosis. Additionally, circulating and urinary C1q levels are not significantly correlated with kidney injury severity, and there is no cross-reactivity between anti-C1q and anti-GBM antibodies (31). The reasons for the absence of C1q deposition are unclear. a-C1q Abs have been shown to promote the accumulation of C1q in target organs and on the surfaces of cells. This may be due to two reasons. Firstly, the levels of a-C1q Ab in circulation are mostly low, which weakens their effect on C1q deposition. Secondly, unlike C3d and C4d, C1q does not covalently bind to its ligands, thus having a short half-life in the body and being easily cleared by macrophages (32).

In all patients with anti-GBM GN, linear and/or granular deposits of C1q, factor B, properdin, C3d, C4d, and C5b-9 can be detected in the glomeruli. C1q, factor B, and properdin co-localize with C5b-9, while properdin co-localizes with C3d. Deposition of factor B and C5b-9 is significantly greater in glomeruli with a crescent formation than in those without. Mannose-binding lectin (MBL) shows diffuse deposition in the mesangium, GBM, Bowman’s capsule, and crescents; does not co-localize with C5b-9; and only partially co-localizes with C4d, suggesting that the LP may not be involved in complement activation in human anti-GBM disease. The reason is that in human anti-GBM GN, the complement system is generally activated through the AP and the CP. The AP may play an important role in complement activation-induced renal damage (33). C4d is a by-product of the CP and LP, and therefore, in cases of immune complex-mediated GN involving the CP and LP, the deposition of C4d will be noted. Because of its thioester bond, it can covalently bind to cell surfaces and serve as a marker for complement activation. For anti-GBM GN, the most common deposition site of C4d is the glomerular capillary wall, which mostly activates the CP (34). However, there have been reports that circulating anti-GBM antibodies are primarily of the IgG4 subclass (35). While the IgG4 subclass are generally regarded as having a diminished capacity for complement activation due to their inability to bind to C1q, they retain the ability to activate the lectin complement pathway through their interaction with MBL. This interaction can subsequently trigger the complement system, resulting in substantial deposition within the renal tissues (36). Complement involvement in anti-GBM GN is reflected not only in renal pathology. Researchers have found that 15% and 100% of patients have elevated levels of C5a in plasma and urine, respectively, while 30% and 92% of patients show increased levels of soluble C5b-9 (SC5b-9) in plasma and urine. Additionally, plasma SC5b-9 and urine C5a levels are positively correlated with baseline serum creatinine levels and the proportion of crescents. Therefore, the pro-inflammatory action of complement C5a and/or the cell lytic action of C5b-9 plays a pathogenic role in anti-GBM GN. Both can be used as indicators for clinical monitoring and predicting disease prognosis (37).

## Laminin-521 and complement in anti-GBM GN

Laminin-521 (LM521) has recently been identified as a novel autoantigen for anti-GBM disease (7). LM521 can induce anti-LM521 antibodies (Abs), leading to the pathogenesis of anti-GBM GN (38). Anti-LM521 autoantibodies (autoAbs) are specific for anti-GBM/GP diseases and are not detected in other glomerular diseases. The positivity rate (10%–38%) of anti-LM521 autoAbs in anti-GBM GN patients is comparable to that of anti-MPO autoAbs (39, 40). Among 101 Chinese patients, 33% were positive for LM521 autoAbs. The presence of LM521 autoAbs is associated with younger age, hemoptysis/pulmonary hemorrhage, and severe hematuria. Importantly, the presence of LM521 autoAbs is associated with worse prognosis, including a higher incidence of reaching the composite endpoint of ESKD or death (40). Anti-LM521 autoantibodies mainly consist of IgG1 and IgG4, which may be involved in tissue damage through different effector mechanisms. Tissue-bound anti-LM521 IgG1 can induce inflammation by activating the complement system (40). Complement FH-related protein 5 (CFHR5) is a surface complement activator that acts in conjunction with LM521. FHR5 deposition can be detected in glomeruli under pathological conditions, and it co-localizes with LM521 in diseased kidneys (41). However, the precise mechanism by which LM521 activates complement and contributes to anti-GBM GN pathogenesis remains unclear and requires further investigation.

## Heparan sulfate proteoglycan and complement in anti-GBM disease

In addition to α345(IV) collagen and laminin 521, heparan sulfate proteoglycan (HSPG) is also a major component of the GBM. The main component of HSPG, heparan sulfate (HS), regulates local complement activation by recruiting complement regulatory protein FH. FH selectively inactivates C3b bound to host HS, thereby limiting complement activation on the GBM (42). By day 10 after inducing anti-GBM GN in mice, glomerular heparanase levels had increased, coinciding with infiltration of endothelial, epithelial, and inflammatory cells into the glomeruli. Following administration of effective anti-heparanase polyclonal antibodies, proteinuria in mice significantly decreased (43). Thus, an intact HSPG structure capable of binding FH is essential for regulating complement activation during the progression of anti-GBM GN.

## Treatment of anti-GBM GN

Anti-neutrophil cytoplasmic antibody (ANCA)-associated glomerulonephritis (AAGN) and anti-GBM GN share similarities in pathogenesis and therapeutic strategies. Several studies suggested that AAGN may initially progress according to its typical disease course, after which anti-GBM antibodies could appear along with related clinical symptoms (44–46). The release of ANCA can damage the kidneys, which may expose a3 (IV) NC1. This exposure leads to the infiltration of CD11c^+^ macrophages, and the exposed GBM epitope can trigger the production of anti-GBM antibodies (46).

The rapid removal of circulating antibodies is essential for effectively treating anti-GBM GN, along with immunosuppressive therapy to reduce the production of autoantibodies. The introduction of plasma exchange has significantly improved the prognosis for patients with anti-GBM GN (47). According to the 2021 KDIGO Glomerular Diseases Guidelines, plasma exchange should continue until anti-GBM antibody levels are no longer detectable (48). In addition to using plasma exchange to eliminate circulating antibodies, immunosuppressive treatments that further inhibit antibody production are fundamental to managing anti-GBM disease (48). The traditional treatment strategy typically includes a combination of corticosteroids and cytotoxic drugs like CTX.

## Complement pathway as a therapeutic target for anti-GBM GN

In the treatment of anti-GBM illness, complement activation is crucial, and targeted therapy that targets the complement system is anticipated to emerge as a novel therapeutic approach. Eculizumab is a humanized monoclonal antibody targeting C5, which can block the cleavage of C5 and inhibit the formation of C5a and C5b-9. It has been approved for the treatment of complement-related diseases. Recent case reports have shown that eculizumab can also be effective as a salvage therapy in patients with anti-GBM disease. In two cases of anti-GBM disease, after treatment with glucocorticoid pulses, CTX, and other treatments, renal function continued to decline. However, after treatment with eculizumab, their renal function improved, and the anti-GBM antibodies have disappeared and have remained normal (49). In two cases of Goodpasture’s syndrome, eculizumab treatment blocked complement-driven lung injury, leading to improved lung function (50, 51). There are relatively few reported cases of eculizumab treatment for anti-GBM GN. After eculizumab treatment, the anti-GBM titers reach background levels within 16–20 days (49). However, long-term use of eculizumab in renal disease is not a panacea; there is a significant (550-fold) increase in the risk of meningitis and risks of other pyogenic infections. While eculizumab is generally considered safe, additional vaccination is required to mitigate these infectious risks (52). Currently, no large-scale clinical trials have evaluated complement inhibitors in anti-GBM disease, and higher-level evidence is needed to establish their safety and efficacy. In addition, some anti-GBM GN patients experience disease recurrence after kidney transplantation due to the reactivation of anti-GBM antibodies despite immunosuppressive therapy (53). The potential impact of bevacizumab on reducing anti-GBM levels either partially or completely to lower the recurrence rate following kidney transplantation is a topic that warrants investigation and consideration.

## C5a receptor antagonist: avacopan

In the common pathway of complement activation, C5 is cleaved by C5 convertase into C5a and C5b, with C5b subsequently initiating the formation of the MAC. C5a has two receptors, C5aR1 (CD88) and C5aR2 (C5L2), both of which are 7-transmembrane receptors that bind C5a with high affinity. Although C5a interacts with both receptors, the majority of it still mediates pro-inflammatory and immunomodulatory effects on the organism. Avacopan (CCX168), an antagonist of C5aR1, has demonstrated effectiveness in treating AAV and has emerged as a new therapeutic option, serving as a novel anti-complement medication to help manage inflammatory diseases (54). The combination of avacopan with PE, CTX, and RTX has demonstrated favorable clinical efficacy in patients with crescentic GN who are double-positive for both ANCA and anti-GBM antibodies; avacopan has shown particular effectiveness in promoting the recovery of renal function (54, 55).

## Conclusion

In general, the presence of C3 alone in glomeruli indicates AP activity; the presence of C4 and C3 without C1q indicates LP activity, which may be related to AP-dependent amplification; and CP activity can be detected by measuring C3, C4, C1q, and IgG in glomeruli (56). Anti-GBM GN appears to be associated with all three complement activation pathways, posing a challenge to clinical therapies aimed at inhibiting complement activation. Complement inhibitors have been approved for kidney diseases such as aHUS and AAV, and are currently being tested for many other kidney diseases. In individuals with deteriorating kidney function, these investigations have shown that complement inhibition is both safe and effective. However, the full potential and limitations of complement inhibition in treating kidney diseases remain unknown. Anti-GBM GN is a very rare disease and none of the currently available therapies are validated according to evidence-based medicine (EBM) principles. However, for anti-GBM patients presenting with severe renal injury, the use of complement inhibitors represents a critical therapeutic intervention.
